# The Feeble-Minded and Their Care

**Published:** 1909-08-07

**Authors:** G. H. Savage

**Affiliations:** Consulting Physician for, and Lecturer on, Mental Diseases at Guy's Hospital


### Hospital Clinics.

### THE FEEBLE-MINDED AND THEIR CARE.

By G. H. SAVAGE, M.D., F.R.C.P.; Consulting Physician for, and Lecturer on, Mental Diseases at Guy's Hospital.

(Abstract of a lecture delivered at the Medical Graduates' College and Polyclinic.)

I feel that the subject of my lecture to-day is both a medical and a social one, and that it is of the greatest importance. No doubt a good many, perhaps all, of you have seen the report, in eight large volumes, of the Commission on the Feeble-Minded, which has recently sat. The summary of the whole, which occupies the eighth volume, is especially important; and I am myself particularly interested in it because I was asked to contribute an analysis of it to the *Quarterly Review*.

The Commissioners begin by accepting the definition of the Royal College of Physicians, according to which a feeble-minded person is one who is capable of earning a living under certain favourable circumstances, but is incapable by reason of mental defect of managing his affairs with ordinary prudence. I must point out here the all-importance of early recognition of the disorder, and of long-continued treatment. Control and treatment must be kept up as long as is needed. One of our great difficulties is that when the insane reach the level of reason they are discharged as cured, and so get beyond the reach of treatment just when it ought to be persevered with. One might just as well argue that when the sufferer from typhoid fever reaches a normal temperature he is then and there fit to get up and go about his business. The feeble-minded should not be returned to the stress of the world until it is certain that they are really fit to hold their own in it.

It is, further, to be desired that the care and control of the feeble-minded should fall under one authority and one only. This is, perhaps, a counsel of perfection at present. We are hardly advanced enough socialistically—or possibly one ought to say autocratically—to put on one side everyone who is not perfectly sane. For, first of all, it is to be considered exactly who the feeble-minded are. Every person above the grade of idiot or imbecile up to those who are nearly normal is classed as being feeble-minded. Certain of these ought decidedly to be put on one side—the inebriates, the criminals, those with immoral tendencies—and their control and treatment ought to be continued as long as it is needed, which in the case of the inebriate is generally for life. Also the epileptics must as a class be said to be feeble-minded, and they too must be included in the scheme. It is a great point to prevent these types from propagating. Yesterday, when I was preparing this lecture, I could not help noticing in front of me an advertisement of a book from Cambridge on the evolution of the vertebrates, and, naturally enough, I began to think of the evolution of the invertebrates, these backboneless people whom it is of the utmost importance to segregate.

From a medico-legal standpoint the first consideration that arises is that these patients are not certifiably insane. In filling in the present insanity certificates one has to put in symptoms observed by oneself; and in the majority of the feeble-minded there is nothing that can be put down on a certificate as a symptom of insanity observed by oneself. When the feeble-minded are properly cared for, some form of certificate may be obtainable which will cover these cases, but at present no certificate of insanity can be given.

There are two main divisions of the feeble-minded: the intellectually weak who are weak all round, are often backward as children, and are permanently affected throughout life; and those whose defect is more an inability to direct and co-ordinate such intellect as they possess. Already large provision is being made for the education of the physically crippled: Lord Mayors take up the matter and found homes for them; but for the morally defective very little provision is made other than in prisons and reformatories. Then there is the large group of those who are often called "wasters," and are allowed to run at large. It is not uncommon for me to be consulted about some young man whose grandfather and father may have piled up a huge fortune in business, but who is himself chiefly engaged in scattering the heap. This of itself would not matter did not injury to others too often accompany the process. The waster is a cause of harm and damage to others, but how we are going to secure him individually I hardly know.

The same is true of inebriates. It is perfectly certain that some inebriates can never be cured, do what one will, and ought to be put on one side. As for epileptics, there are, of course, epileptics and epileptics. It has been said that certain great geniuses, such as Napoleon and Mohammed, have been epileptics, and there is no doubt that some of the occasional or casual epileptics are brilliant men, to put aside whom would be very unjust. But those epileptics who are constantly subject to the disorder should be prevented from having offspring.

Among the difficulties of the question, there is the disposal of a large group of those who come into the world handicapped, and never get strong; they are often subject as children to fits or rages on very slight provocation. Others get as far as adolescence or maturity before they break down and become weak-minded. They are like eight-day clocks, which can only be wound up to go for a certain space of time and no longer.

Another point which the Committee seems to have hardly grasped is that if we seclude all those who are likely to add to the insane population if they marry, there are to be considered those who after a mental breakdown may recover and remain well for one, two, three, four or more years, but who will, it is perfectly certain, ultimately break down again. These patients are perfectly reasonable during the intervals, and they cannot be considered as being permanently weak-minded.

Not only is direct heredity from acute insanity in the parent dangerous; this danger is comparatively slight beside the prospect of an epileptic or weak-minded person having defective offspring. Not every acute lunatic begets children who are mentally deficient. It is well to remember that there is a much greater danger of transmission of apparently slight deviations from the normal. This is strictly in accordance with the principles of Darwin. The degenerating or epileptic father is more likely to beget feeble-minded children than is even he who has been acutely insane. So, too, the child begotten by a general paralytic stands a very great chance of being permanently weak-minded or insane. The effect of a tuberculous parentage is not quite the same: but it stands to reason that the child of anyone whose general health is seriously impaired by tuberculosis is not likely to be robust, and may be defective mentally as well as bodily.

The question is often asked: Is simple consanguinity likely to produce feeble-mindedness? Usually it is in connection with the marriage of cousins that this is put. The parents are, as a rule, dead against such marriages. Now, the truth of the matter is that if there is neurosis or actual insanity in either family, it is a serious matter, and marriage should be forbidden. But apart from that, simple consanguinity is of itself of much less importance in the production of mental deficiency than is generally supposed.

The feeble-minded are often described as being easily led, as wanting in will-power and in adaptability. Anything of the nature of epilepsy is often thus represented in the next generation. A neurotic child, too, who might otherwise develop all right, may suffer from some physical disorder in childhood, and may then be affected by some degree of feeble-mindedness. A series of convulsions or a febrile attack of any kind may act in this way. The frequency with which interference with respiration causes mental enfeeblement is very interesting to me; measles and whooping-cough may act thus. There is also no doubt that certain individuals owe their feeble-mindedness to drugs or to injuries; of the former especially to opiates and to gin administered to produce sleep. I see not uncommonly young giants, boys of 15 or so, who stand 6 ft. 3 in. or 6 ft. 4 in., and have outgrown their nervous systems. Unfortunately, the majority of these never recover completely.

Let us take an example of the first class into which I divided the feeble-minded, those who are intellectually weak all round. A child is born apparently healthy, and all goes well until it is about 18 months old, when the mother notices that the baby neither walks nor talks. Another six or 12 months may go, and the child then begins to totter about, though it is often able to crawl actively at the usual age, but still it does not talk; it may not begin to do this until it is three or four years old. These are the most important cases of defective development likely to end in permanent feeble-mindedness. Such children are very subject to rages; little things upset their balance; night terrors are not infrequent, and wetting of the bed is common. They are, in fact, long in being taught the ordinary conventions of society. Yet these children are usually looked upon by their friends as merely backward. They may in time learn a certain amount of truth and honesty, and even master some book learning. I am told sometimes about a child which has not learned to walk until four years old that it is very musical. I ask then precisely what the phrase means, and I generally find out that the child can at once reproduce by humming any tune it hears; but it cannot be taught to play. Such children can reproduce music, but cannot develop. I can visualise while I am talking to you an individual whom I have long watched. At about 14 he was apprenticed to a tailor, and after three years of this he was sent to another situation as an improver. But at the end of 24 hours he was sent home, for it turned out that he was unable to do anything beyond sewing. He was then set to bootmaking, but could never learn anything beyond hammering in a nail. Then it was thought he might best be used running errands, but here too he was useless. Yet he was a nice bright boy, and perfectly willing; but he could be made no use of whatever at anything. I saw a young woman once who was coming into a large property, and was brought to me for an opinion about her fitness to look after it. When I first saw her I thought how very sensible she seemed, and that it was rather a shame to subject her to the stigma of being thought weak-minded. At my request she read some French out of a book, and conversed with me; but on asking her to do some sums she failed utterly. She could not add two and two, or count up a handful of money.

There are very many of these individuals who are at liberty, especially in villages, where they pass muster as "softies." But this class, if allowed to breed, will breed weak-mindedness to a certainty. If these patients can be put to agricultural work, well and good. At one time they were sent into the army or navy, and there is no doubt that the strict discipline and regular life keeps them out of mischief. The establishment of colonies for these folks was considered by the Royal Commission. Now, colonies are all very well, but I have seen another side of the matter. The establishment of such colonies damages property in the neighbourhood. The oldest of all such colonies is at Gheel, in Belgium, the city of the simple; there for 700 or 800 years have been sent the failures from the country round about, and there is there a central receiving depot controlling 15 or 16 villages for the feeble-minded.

A certain number are greatly benefited by religious institutions. A well-conducted convent is often a good place for those easily led people who so often drift into immorality but for some such restraint. The Church Army and other religious institutions do work of the same sort. The whole future of the treatment of the feeble-minded depends on the development of such assistance as institutions and colonies; but success is usually only partial.

Moral defects are much more difficult to deal with. Honesty is a thing that does not come naturally to a child. It has to be taught, and it is a slow acquisition for the infant to discover that everything that can be touched does not belong to it. Nowadays we send the young thief to an institution where he is kept under strict discipline, and a certain number are thus reformed. One does not want punishment to be revenge, but equally it is not desirable that the criminal should feel he is not responsible for his offence. Sexual morality is very much wanting in the feeble-minded. At any of the homes you will find that a large number of the women are the mothers of illegitimate children. Thieves one must punish to a certain extent, but as far as moral defects are concerned segregation is the only way. I was very much astonished to find that in democratic America there is a strong feeling among experts that the man who is convicted three or four times should be imprisoned for life. Certainly public opinion here would not sanction such a proposal.

For inebriates retreats are very useful. There are brands plucked from the burning in all sorts of ways—by drugs, by the Salvation Army, certainly by hypnotism, and by retreats. But we have hardly yet got so far as to seclude all habitual drunkards. Some dipsomaniacs are wonderful men, who it would be a great pity to put away altogether.

Constant occupation is necessary in the education of weak-minded children. Music, drill, games, gymnastics, and a simple outdoor life as far as possible are all important. As long as children are under age one can do what one likes with them; the difficulty is what to make of them as they grow up. Various avocations have been suggested. The present rage is for poultry farming and fruit farming; but there is a limit to both of these, and it must always be remembered that the feeble-minded is one who cannot *unaided* make his own living.

Of the next group, the epileptics, I must speak very shortly. There are now several epileptic school colonies, some of which contain several hundred children who are neither imbecile nor idiotic. These are to a certain extent educable, but will never be more than feeble-minded. First of all they live in houses of only one story, so there are no stairs for them to fall down. Then there are no ponds, railways, or other possible sources of accidents. Their diet is almost purely vegetarian, and they, of course, get no stimulants. They have object lessons, singing, musical drill, and simple teaching of that sort, with a certain amount of bromides, but not much—much less, as a rule, than they have been accustomed to take before joining the colony. Such children thus treated improve in a remarkable way, but they never become normal. They work perfectly well at carpentering or laundry work, etc., as long as they are watched. This group is a very unfortunate one, for when they leave at 16 or so they drift ultimately into workhouses, as they are not fit to be in the charge of their relatives.

One hesitates to condemn every feeble-minded child as feeble-minded for life, because undoubtedly some of them do become normal. But these are very few; the majority never do so. The essential thing for them all is early recognition and early treatment.

